# Free-Form and Deformable Energy Storage as a Forerunner to Next-Generation Smart Electronics

**DOI:** 10.3390/mi11040347

**Published:** 2020-03-26

**Authors:** Soyul Kwak, Jihyeon Kang, Inho Nam, Jongheop Yi

**Affiliations:** 1 School of Chemical Engineering and Materials Science, Institute of Energy Converting Soft Materials, Chung-Ang University, Seoul 06974, Korea; chatte0614@cau.ac.kr (S.K.); kar04114@cau.ac.kr (J.K.); 2 School of Chemical and Biological Engineering, Institute of Chemical Processes, WCU Program of Chemical Convergence for Energy and Environment, Seoul National University, Seoul 08826, Korea

**Keywords:** bendable and foldable batteries, stretchable batteries, flexible materials, flexible configurations, energy storage systems

## Abstract

Planar and rigid conventional electronics are intrinsically incompatible with curvilinear and deformable devices. The recent development of organic and inorganic flexible and stretchable electronics enables the production of various applications, such as soft robots, flexible displays, wearable electronics, electronic skins, bendable phones, and implantable medical devices. To power these devices, persistent efforts have thus been expended to develop a flexible energy storage system that can be ideally deformed while maintaining its electrochemical performance. In this review, the enabling technologies of the electrochemical and mechanical performances of flexible devices are summarized. The investigations demonstrate the improvement of electrochemical performance via the adoption of new materials and alternative reactions. Moreover, the strategies used to develop novel materials and distinct design configurations are introduced in the following sections.

## 1. Introduction

Over the past decade, highly deformable electronics have attracted considerable attention as promising alternatives to conventional rigid electronics. In particular, the electronic components of soft and stretchable devices have remarkably advanced for next-generation, human-friendly applications of electronics, such as wearable electronics, electronic paper, smart clothing, electronic skins, displays, and implantable medical devices. 

Although the demands for future human-integrated devices, transport, and storage of fluctuating energy sources have increased, “energy storage systems” have rarely been improved. As a result, storage has become a limiting factor in the achievement of complete and independent stretchable electronics for the next generation. Conventional batteries are typically rigid and heavy because of the fabrication of coating slurry containing the active material. This process, however, presents certain disadvantages. For example, the metal current collector cannot regain its original shape when subjected to repeated bending and stretching [1]. 

Among other materials, lithium-ion batteries (LIBs), which are lightweight, high in energy density, and low in self-discharge, are among the most ideal candidates for energy storage systems, as they can afford the opportunity for the development of rechargeable applications that are extremely advantageous for portable consumer electronics [2,3,4,5,6,7,8,9,10]. Recent research interests have been focused on flexible lithium-ion batteries with considerably enhanced energy density and cycling stability [11,12,13]. Conventional LIBs, however, preclude their application as support for the next generation of stretchable devices. To resolve these problems, considerable efforts have been expended as follows. (1) Adoption of intrinsically flexible materials for flexible energy storage systems. The use of materials that can be bent, folded, and stretched is a common strategy to achieve flexible electronics. Intrinsically flexible materials afford a direct route with which to achieve higher mechanical robustness, higher device density, and scalable fabrication. Among the recognized stretchable substrate materials suitable for the fabrication of implantable electronic systems are polydimethylsiloxane (PDMS), polyaniline, and carbon-based materials. These have considerable potential because of their specific mechanical properties (compliance and softness), good biocompatibility, and thermal stability [14,15]. Accordingly, these are frequently utilized as substrates, scaffolds, or transfer media for flexible electronics. Specifically, several studies have fabricated uniform substrates made of aforementioned intrinsically flexible materials and mounted other parts, and other studies used those materials as binders to can keep rigid parts from separating. (2) Various design studies have also demonstrated different means with which to enhance the deformability of flexible electronics. Metals are the materials of choice for the majority of existing nanoelectronic devices. In the fabrication of flexible devices, however, it is extremely important to prepare new designs to impart flexibility to rigid metal films. This is because, in general, these materials can withstand a strain of only <1%. Over the years, various design strategies have been employed by different researchers in this regard. Several of these major schemes involve wavy-shape (buckled or wrinkled) designs, serpentine bridge–island designs, and designs inspired from paper, textile, wire shape, origami, and kirigami [16]. This review, therefore, focuses on the different means of introducing flexibility into each component of flexible lithium-ion batteries and other types of batteries via innovative materials and designs. 

## 2. Bendable and Foldable Batteries

Wearable technology and electronic textiles are the major areas of advancement for thin films and flexible batteries. Conventional secondary batteries may satisfy the energy requirements of wearable devices; however, they fail to achieve adequate flexibility, thinness, and lightness of weight. These new market requirements afford the opportunity for energy storage solutions with novel form factors. The majority of thin-film battery companies claim to have ongoing projects in the field of wearable technology. Moreover, a number of investigations for developing flexible electronics have been conducted. In the following section, the strategies that have been implemented to provide energy storage devices with bendability and foldability are discussed. 

### 2.1. Strategies for Achieving Bendability and Foldability: Materials

#### 2.1.1. Graphene

Graphene has excellent electrical conductivity and flexibility, a large contact area with electrolytes, and sheet-like nanostructure with a short diffusion pathway for lithium, which promote the reaction kinetics of the composite [17]. For long-term battery cycling in conventional batteries, the low adhesion with oxide materials would be problematic. To solve this problem, Gwon et al. proposed a graphene-based hybrid electrode for a new path using a V_2_O_5_/graphene cathode and graphene anode that offers better compatibility and high adhesion with cathode materials. No change in the charge/discharge capability is exhibited, even when the battery is deformed [18]. 

**Figure 1:**
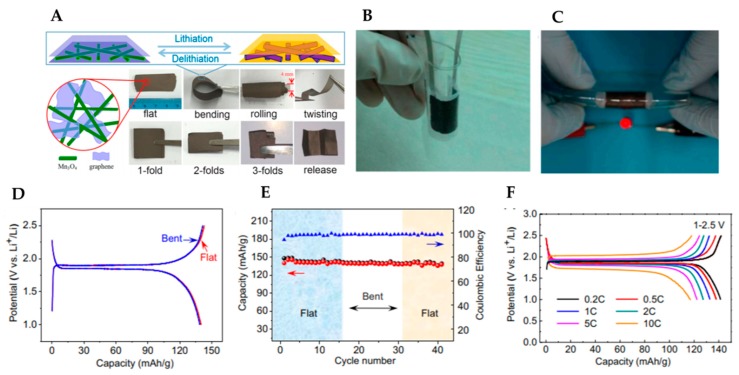
(**A**) Schematic (top view and cross-sectional view) of rGO/Mn3O4 membrane and its digital photographs illustrating the flexibility (bending, rolling, and twisting) and foldability (1, 2, and 3 folds) states. Adapted with permission from [27]. Copyright (2014) *Advanced Materials*. Characterization of a thin, lightweight, and flexible LTO/GF or LFP/GF full battery. (**B**) Photograph of a bent battery encapsulated by PDMS, exhibiting good flexibility. (**C**) Lighting a red LED device at a bent state. (**D**) Galvanostatic charging/discharging curves of battery. Red and blue lines represent the as-fabricated flat battery and bent battery after 20 times of repeated bending at a 5-mm radius, respectively. (**E**) Cyclic performance of the battery in flat and bent states. (**F**) Charging–discharging voltage curves of battery with different current rates. Adapted with permission from [28]. Copyright (2012) *Proceedings of the National Academy of Sciences.*

The reduced graphene oxide (rGO) material is a flexible freestanding porous carbon film with a high specific surface area [19]. Wang et al. fabricated an rGO/Mn_3_O_4_ membrane that can sustain various mechanical deformations, such as bending, rolling, twisting, and folding [20]. Figure 1a illustrates the classical membrane structure in which long one-dimensional (1D) Mn_3_O_4_ nanowires and two-dimensional (2D) rGO nanosheets are integrated via a simple vacuum filtration method to create a robust 1D/2D hybrid architecture. In this architecture, 1D nanowires are intertwined with graphene nanosheets, thereby producing strong synergy. The nanowires can serve as spacers that prevent the adjacent graphene from restacking and agglomerating, thereby expanding the electrode/electrolyte interface. Moreover, graphene can function as a strain cushion to reduce the large volume change of Mn_3_O_4_ and act as a conductive network to increase electron flow throughout the electrode. The nanosize structures can reduce electron/Li^+^ transport distances for improved reaction kinetics. As a result, the flexible rGO/Mn_3_O_4_ nanocomposite can yield a high rate capability and cycling stability.

Apart from 2D graphene, 3D graphene (e.g., foam, aerogel, and hydrogel) has been developed to overcome the problems presented by conventional batteries. These batteries do not exhibit adequate flexibility because of the weak bonds between the electrode material and current collector; consequently, they tend to detach after repeated bending. Moreover, the kinetic limitation of lithium-ion diffusion through an electrode material on the current collector generates a considerable electron and ion transport resistance and results in the loss of fast charge/discharge rate capability. To solve these problems, numerous studies have been conducted in recent years to convert graphene into a 3D network [21]. Such a network exhibits a large surface area, better permeability, and wider active sites [22,23,24,25]. The advancements in LIB technology are expected to be achieved by combining a flexible design approach and a hierarchical 3D network [26]. 

The use of graphene foam (GF) is extensively investigated for flexible energy storage devices because of its high conductivity, light weight, high specific surface area, and excellent flexibility [27]. It also has high porosity (approximately 99.7%) and can be bent to arbitrary shapes without breaking. The GF network is used as a highly conductive pathway for electrons, ions, and a 3D interconnected current collector. Chen et al. have developed a flexible GF with Li_4_Ti_5_O_12_(LTO) and LiFePO_4_(LFP) as the anode and the cathode, respectively [28]. The LFP/GF and LTO/GF electrodes exhibit high capacity and high rate performance. They are capable of tolerating repeated bending to a radius of less than 5 mm without undergoing structural failure or loss of performance. This flexible battery can power a red light-emitting diode (LED) at a bent state, as shown in Figure 1b,c. After bending the battery at a 5-mm radius for 20 times, only a negligible overpotential is observed, and the capacity of the bent battery decreases to less than 1% of the original flat battery capacity, as shown in Figure 1d. This flexible battery exhibits excellent cyclic stability both under flat and bent states. It exhibits capacity retentions of approximately 97% and 95% of the original after the first 15 bending cycles in a flat state and another 15 cycles in a bent state (5-mm bend radius), respectively, as shown in Figure 1e.

**Figure 2:**
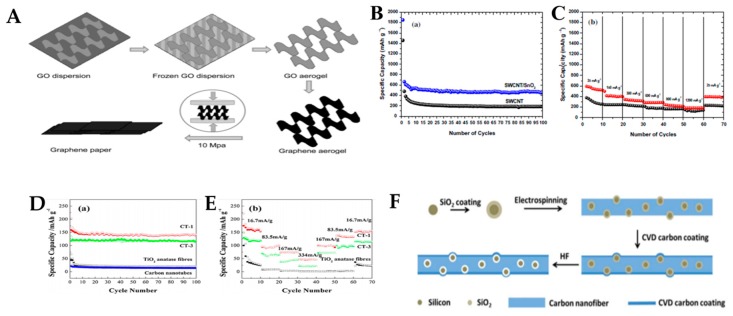
(**A**) The illustration is the formation process of graphene paper. The first step is freezing GO aqueous dispersion at −50 ℃ to obtain GO aerogel, the second step is freeze-drying under vacuum, the third is obtaining graphene aerogel by treating GO aerogel at 200 ℃ in air, and the last step is the mechanical pressing of the graphene aerogel to form graphene paper. Adapted with permission from [29]. Copyright (2012) *Adv Mater.* (**B**) Cycling stability of SWCNTs and SWCNT/SnO_2_ anode paper at a constant current density of 25 mA g^−1^. (**C**) High rate capability of the SWCNT/SnO_2_ anode paper. Adapted with permission from [31]. Copyright (2012) *Carbon*. (**D**) Galvanostatic tests of CNT/TiO_2_ composites (consisting of CT-1, CT-3, and TiO_2_ nanofibers, carbon nanotubes) at 16.7 mA/g current density between 1 and 3V (vs. Li/Li^+^). (**E**) Multi-current density galvanostatic tests of CNT/TiO_2_ composites (consisting of CT-1, CT-3, and TiO_2_ nanofibers) at 16.7–334 mA/g between 1 and 3V (charge/discharge). Adapted with permission from [32]. Copyright (2013) Adv Mater. (**F**) Schematic of the fabrication process for the vacant Si@CNF@C composite. Adapted with permission from [35]. Copyright (2014) *Nanoscale*.

Graphene aerogel is another key material for constructing 3D structures. Liu et al. reported the use of graphene aerogel to build a foldable structured graphene paper. The aerogel is prefabricated by freeze-drying a GO aqueous dispersion followed by thermal reduction [29]. The formation process of graphene paper is shown in Figure 2a. In this type of paper-like carbon material, layer folding and stacking exist. When used as a LIB electrode, this material is flexible, binder-free, free-standing, and suitable for mass production; when tested as a LIB anode, it exhibits a high charge capacity of 864 mAh∙g^−1^ with Coulombic efficiencies exceeding 98% starting from the second cycle. 

Despite the abovementioned advantages of graphene, problems persist. Graphene electrodes suffer from large irreversible capacity loss, low initial Coulombic efficiency, and fast capacity fading. These are mainly because of the restacking of graphene sheets and the side reactions between graphene and electrolytes that arise from the functional groups and defects of graphene [11]. In view of these disadvantages, another alternative, carbon nanotube (CNT)-based electrode, has also been investigated.

#### 2.1.2. Carbon Nanotube (CNT)

The CNT network may be employed not only as an excellent electrical substrate, but also as a porous matrix that buffers the volume expansion of anode materials during lithiation. This network can thus minimize the internal stress in the electrode and preclude the delamination of the electrode coating and current collector [30]. Single-wall carbon nanotubes (SWCNTs) are fabricated by folding a graphene sheet to form a cylinder. These are among the most promising materials for LIBs because of their excellent physical properties, such as high flexibility, high theoretical tensile strength, and high electrical conductivity. Using these nanotubes, Noerochim et al. developed a SWCNT/SnO_2_ paper that can prevent substrate damage or peeling during bending [31]. This type of paper not only significantly improves the cycling performance of high-capacity electrodes but also accommodates considerable volume changes.

In the SWCNT/SnO_2_ paper, CNTs form a 3D nanoporous network structure, with SnO_2_ particles deposited onto the SWCNT surface at selected sites. The paper exhibits a high specific discharge capacity of up to 454 mAh∙g^−1^ at a current density of 25 mA∙g^−1^ and stable cycling stability of up to 100 cycles, as shown in Figure 2b. The high rate capability of the free-standing binder-free SWCNT/SnO_2_ electrode upon cycling is demonstrated in Figure 2c. Bending the SWCNT/SnO_2_ electrodes into small radii of curvature has a minimal effect on the electrochemical behavior, reflecting a small increase in their electrical resistance. Zhang et al. also investigated the flexible and free-standing CNT and TiO_2_ nanofiber composite film by employing the flow-directed assembly method without the use of binders/conventional copper current collectors [32]. These binder-free electrodes possess the advantages of a supercapacitor; they can bend smoothly and operate at high temperatures (even of up to 200 °C). The CNT can prevent the agglomeration of TiO_2_ nanofibers and improve the lithium-ion conductivity between the electrolyte and TiO_2_ nanofiber active material. TiO_2_ is used as an anode material of lithium-ion batteries because of its low volume expansion upon lithiation and its charge/discharge capacity at a high current density [33,34]. At a 16.7-mA∙g^−1^ current density, CT-1 exhibits a 200-mAh∙g^−1^ discharge capacity at the initial cycle and a reversible capacity of approximately 150-mAh∙g^−1^ up to 100 cycles. Discharges capacities of 135-mAh∙g^−1^ in the first cycle and approximately 125 mAh∙g^−1^ after the second cycle are exhibited by CT-3 (Figure 2d; meanwhile, the CT-1 and CT-3 have ratios of pure carbon nanotubes to TiO_2_ nanofibers of 1:1 and 1:3, respectively). These data demonstrate that CNT networks are effective in enhancing lithium-ion storage capacity and improving the cycle stability of TiO_2_ nanofibers. In contrast, pure TiO_2_ nanofibers exhibit sharp capacity decays in the first 20 cycles (i.e., from more than 200 mAh∙g^−1^ at the initial cycle to approximately 25 mAh∙g^−1^ after 20 cycles). Figure 2e shows that CT-1 and CT-3 have considerably higher rate capacities than pure TiO_2_ nanofibers at the same current density. Moreover, the composite with a higher CNT ratio (i.e., CT-1), especially at a higher current density, exhibits a higher capacity than that with a lower CNT ratio (CT-3). These results indicate that TiO_2_ nanofibers can be easily incorporated into a CNT network, and larger CNT ratios are advantageous for electron collection during the lithium-ion intercalation/extraction processes in TiO_2_. As a result, the CNT–TiO_2_ composite membrane exhibits remarkable electrochemical performance in terms of reversible capacity and rate capability.

#### 2.1.3. Carbon Nanofibers (CNFs)

In addition to CNTs and graphenes, carbon nanofibers (CNFs) have attracted considerable interest in electrochemical energy storage, especially in flexible energy storage because of their low cost, rapid production rate, and varied structural designs [30]. Extensive studies based on CNFs have been conducted to solve the expansion problem of active materials (e.g., Si), fabricate free-standing electrodes, and realize flexible and bendable batteries [35,36,37]. Fu et al. applied additional carbon coating to the vacant Si@CNF structure to suppress the reversible formation of a solid electrolyte interface within the CNF structure [35]. It can endure several cycles of bending without any evident fracture. The nonwoven mat can be directly used as an electrode without a current collector or polymer binder. Although pure carbon nanofibers exhibit a higher charge capacity of 300 mAh∙g^−1^ at the 50th cycle, the capacity of vacant Si@CNF@C lies in the range of 780–612 mAh∙g^−1^ in the first several cycles and then remains at 620 mAh∙g^−1^ until the 200th cycle. It, therefore, demonstrates an enhanced capacity over pure carbon. The vacant Si@CNF@C composite shows significant improvements in cyclic stability, Coulombic efficiency (>99%), and capacity retention (80%), indicating that Fu et al. successfully solved the volume expansion problem by adopting this method. 

#### 2.1.4. The Other Materials

Other than carbon-based conducting materials, there have been massive developments in conducting polymers with unique properties that can meet the recent needs in various areas. Well known conducting polymers, including polyacetylene (PA), polyaniline (PANI), polypyrrole (PPY), poly(p-phenylenevinylene) (PPV), poly(3,4-ethylene dioxythiophene) (PEDOT), polyfuran (PF), and other polythiophene (PTh) derivatives, can reverse the doping/dedoping process and they have great processability [38]. The aforementioned conducting polymers can be synthesized using electrospinning, hard physical template-guided synthesis, soft chemical template synthesis, and a variety of lithography techniques. A variety of conducting polymers are used in the fields of chemical sensors and biosensors, field-effect transistors, field emission and electrochromic display devices, separation membranes, and energy storage systems [39,40]. A recent study done by Xiao et al. demonstrated a flexible and freestanding film Li-S battery with a sandwich structure between graphene and poly(3,4-eth-ylene-dioxythiophene): poly(styrenesulfonate) simply by vacuum filtration, wherein nano sulfur is homogeneously coated by graphene and PEDOT:PSS [41]. Although Li-S batteries are one of the more promising states of art batteries, challenges still remain: low cycle stability and volumetric capacity. Herein, a highly conductive and compact PEDOT:PSS-graphene network utilizes charge transportation, which results in better sulfur utilization and high-rate performance. Furthermore, flexible graphene and PEDOT:PSS grant Li-S batteries the ability to withstand harsh volumetric changes during the charge/discharge process. Among the conducting polymers, polyaniline (PANI) attracted great interest in energy storage devices for its significant specific capacitance, which is due to multi-redox reactions, and low-cost production process [42]. What is more intriguing is that PANI can be synthesized with various simple methods, in various forms (powder or thin-film). Ma et al. fabricated flexible cathodes by in situ growth of nanostructured PANI on cellulose papers along with flexible Zn-grown graphite papers. Herein, a gel electrolyte is used to fabricate a solid-state flexible aqueous Zn-ion battery [43]. Nanostructured PANI derived flexible cathode was grown on lens papers via in situ polyperization, while the flexible anode was prepared by electrochemically depositing Zn-grown graphite papers at a constant voltage of 0.8V. The Zn-ion battery showed outstanding electrochemical performance, comparable to those of aqueous batteries, or even superior to. Surprisingly, the Zn-ion battery exhibited insignificant changes in electrochemical performance after 1000 bending cycles. Together, conducting polymers are viewed as promising candidates for the new materials for the future flexible electrochemical devices. 

### 2.2. Strategies for Achieving Bendability and Foldability: Designs

#### 2.2.1. Paper Configuration

Paper, invented more than 2000 years ago and presently used widely in daily life, is being explored as a platform for energy-storage devices. This is because, as imprintable electrolytes, cellular paper substrates are highly stable and demonstrate folding characteristics [44,45]. Paper is therefore a potential base material for flexible energy storage devices with combined superior properties, such as high mechanical strength, wide surface area, and excellent electrical conductivity. Moreover, paper has a better lifecycle compared to non-flexible conventional electrode architecture. Essentially, paper-based flexible electrodes exhibit significantly improved performance swith superior specific energy, energy density, and power density—similar to those achieved using solid-state electrolytes [18]. The surface roughness and porous structure of paper-based LIBs make them ideal for ion transportation, and the improved flexibility of these materials also allows them to be used as fully bendable batteries [27]. The following example is related research on the paper-based configuration. 

**Figure 3:**
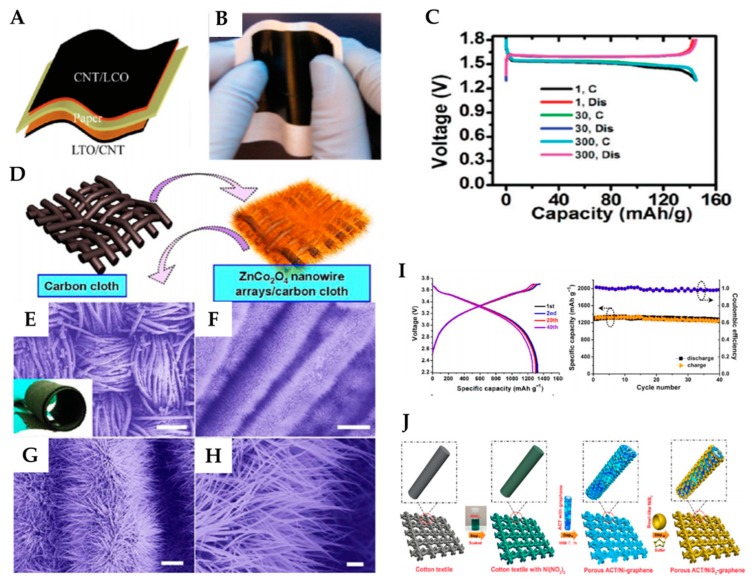
(**A**) Schematic of the final paper Li-ion battery device structure, with both LCO/CNT and LTO/CNT laminated on both sides of the paper substrate. (**B**) Picture of the Li-ion paper battery before encapsulation for measurement. Adapted with permission from [48]. Copyright (2010) *American Chemical Society*. (**C**) Galvanostatic charging/discharging curves of the LTO anode (1.3–1.7 V) half cells with conductive paper current collectors. The mass of the LTO electrode is 1.8 mg. The current rate is C/5. Adapted with permission from [48]. Copyright (2010) *American Chemical Society*. (**D**) Schematic illustration of the synthesis of flexible 3D ZnCo_2_O_4_ nanowire arrays/carbon cloth. (**E**−**H**) Typical FE-SEM images of the ZnCo2O4 nanowire arrays growing on carbon cloth at different magnifications. (Inset in panel e) The photographic image of the product exhibited very good flexibility, and it can be rolled up periodically with a tweezer. Scale bars: 200 μm (**E**); 20 μm (**F**); 5 μm (**G**); 1 μm (**H**). (**I**) Charge−discharge curves for first, second, 20th, and 40th cycles, and cycling performance of flexible full battery up to 40 cycles at a current density of 200 mAh g^−1^. Adapted with permission from [50]. Copyright (2012) Nano Letters. (**J**) Schematic illustration of the fabrication process of porous ACT/NiS_2_−graphene composite. Adapted with permission from [49]. Copyright (2015) *American Chemical Society.*

Wang et al. have reported the manufacture of batteries that combine a cellulose complex with aligned CNT composite paper electrodes [46]. The composite CNT could be twisted to be a flexible composite fiber electrode [1]. Using a nanocomposite paper and a thin evaporated Li-metal layer as cathode and anode, respectively, the assembled flexible paper battery exhibits a reversible capacity of 110 mAh∙g^−1^, which did not change even after it was severely folded [27]. This composite paper exhibits excellent flexibility and can directly be used as a flexible electrode of paper batteries as it can be rolled, twisted, and even bent at an angle, but also completely restored [47]. Hu et al. fabricated the laminated Li-ion paper battery composed of the CNT/LTO/paper/LCO/CNT structure. The CNT thin films are coated onto stainless steel substrates based on a solution process; thereafter, electrode materials are applied [48]. The double layer of LTO/CNT or LCO/CNT can be easily peeled off because CNTs interact only weakly with metal substrates. To enable the double-layer film to adhere to the paper, wet PVDF is used as glue. In this battery, the paper functions as both the substrate and separator, and the highly conductive CNT films function as current collectors for electrodes. The Li-ion paper battery scheme and final device prior to encapsulation and cell testing are presented in Figure 3a,b, respectively. In addition to performing mechanical tests, Hu et al. applied electrochemical tests to compare the 30 and 300-cycle voltage profiles and found that these do not differ, as shown in Figure 3c. The CNT/LTO electrodes achieve an initial discharge capacity of 147 mAh∙g^−1^ and exhibit capacity retention of 95% after 300 cycles at C/5. The paper’s stability has been maintained for eight months in the Li-ion battery test, where the electrolyte is the same as that employed in this study [48].

#### 2.2.2. Textile Configuration

As the most widely used natural fibers for soft and breathable clothing, the textile configuration has been proved to be a remarkable platform for constructing flexible energy storage devices [49]. Liu et al. demonstrated the synthesis of hierarchical 3D ZnCo_2_O_4_ nanowire arrays/carbon cloth for its use as an integrated electrode for flexible LIBs [50]. This cloth is used as a new type of binder-free anode and current collector to replace conventional 2D metal current collectors. The structure of the as-synthesized hierarchical ZnCo_2_O_4_ nanowire arrays/carbon cloth is defined using scanning electron microscopy (SEM), as shown in Figure 3. An effective approach to growing 3D ZnCo_2_O_4_ nanowire arrays with high density into carbon cloth is to use a facile hydrothermal route. The growth process is illustrated in Figure 3d, and Figure 3e–i clearly displays the well-established fabric structure of the ZnCo_2_O_4_ nanowire arrays grown on the carbon fiber cloth. In the study, the structure of the fabricated flexible full battery consists of flexible ZnCo_2_O_4_/carbon cloth, flexible separators, LiCoO_2_/Al foil, LiPF6-based electrolyte, and a flexible plastic shell. This battery demonstrates that an ultrathin device with flexible and paper-like features can be achieved. The voltage-capacity profile of a flexible full battery device prepared for 1, 2, 20, and 40 charge/discharge cycles at a current rate of 200 mA∙g^−1^ and a voltage window of 2.2–3.7 V is shown in Figure 3i. The charge/discharge curves represent two planes with an average discharge voltage of 3.4 V. The initial irreversible discharge capacity of the hybrid electrode is approximately 1314 mAh∙g^−1^; the discharge capacity exhibits good reversibility and is stable. The discharge capacities of the device remain practically constant even after 120 cycles of bending, indicating that the electrical stability of the fabricated flexible battery is virtually unaffected by external bending stress. The voltage-capacity profiles of the as-prepared ZnCo2O4/carbon cloth electrodes for the 1st, 2nd, 50th, and 100th charge/discharge cycles are also shown in the figure. 

Hu et al. adapted a 3D porous textile conductor to develop LIB applications [51]. It is fabricated using a plain polyester fiber textile and well-dispersed SWCNT ink in water and employing 1% sodium dodecylbenzenesulfonate to prevent CNT agglomeration. This conductor, with its 3D network architecture, exhibits high electronic conductivity and robust mechanical stability. It has therefore been used to replace conventional metal current collectors in LIBs. The LTO in the 3D textile electrode with a high mass loading exhibits excellent cycling performance, with less than 10% variation of capacity change in 350 cycles. Coulombic efficiency is typically more than 99.5%. The textile can be bent and easily cut into other shapes without the occurrence of delamination of the battery electrode material from the conductive polyester fibers. In addition, the fabrication of electrodes based on a 3D architecture affords large surface area, better permeability, and reduced path length. It is thus considered as a potential approach to obtain LIBs with high capacity and high rate capability. 

Gao et al. developed an activated cotton textile (ACT) containing porous tubular fibers inserted with NiS_2_ nanobowls and enclosed with conductive graphene sheets [49]. The structure of the battery is simply composed of graphene, ACT, NiS_2_, and graphene layers. Figure 3j shows the design and fabrication procedures of NiS_2_ nanobowl−graphene hybrid architectures on porous ACT tubular fibers. This cell has achieved an ultrahigh initial capacity of ∼1710 mAh∙g^−1^ at a rate of 0.01 C, and a high reversible capacity of ∼1016 mAh∙g^−1^ after 400 cycles at a rate of 0.1 C. On comparing the voltage–specific capacity curve at different charge and discharge rates, it has been found that the mechanical flexibility and electrochemical robustness are similar under normal and bent states. A flexible ACT/NiS_2_ lithium-ion battery also demonstrates its practical application for flexible energy storage devices. This battery can be applied to wearables and integrated into flexible textiles for a variety of electronic devices.

#### 2.2.3. Wire-Shaped Configuration

**Figure 4:**
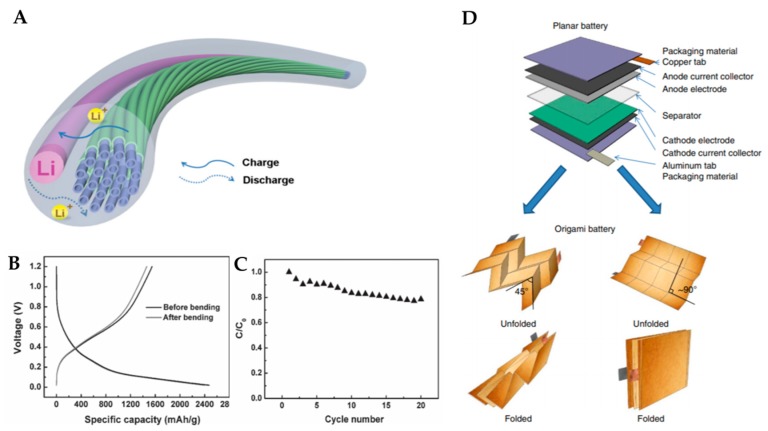
(**A**) Schematic illustration of a lithium-ion battery based on the aligned MWCNT/Si composite fiber as a working electrode. Adapted with permission from [53]. Copyright (2013) *WILEY-VCH Verlag GmbH and Co. KGaA, Weinheim.* (**B**) Charge and discharge curves of a half-cell based on the aligned MWCNT/Si composite fiber with a Si weight percentage of 38.1% before and after bending for 100 cycles at 2 Ag^−1^. (**C**) Dependence of specific capacity on cycle number for the composite fiber after bending for 100 cycles at 2 Ag^−1^. Adapted with permission from [53]. Copyright (2013) *WILEY-VCH Verlag GmbH and Co. KGaA, Weinheim.* (**D**) Schematic illustration of origami lithium-ion battery (LIB). It shows two features of LIBs: (1) Exploded view of the multilayer structure of conventional LIBs in the planar state and (2) two examples of origami LIBs using Miura folding. Adapted with permission from [54]. Copyright (2014) *Macmillan Publishers Limited.*

Comparable to conventional paper-like batteries, wire-shaped electrodes can be interconnected in textiles and are easy to install in electronic devices [52]. In this section, the development of wire-shaped lithium-ion batteries aligned with multi-walled carbon nanotube (MWCNT)/Si composite fibers as anodes for flexibility is presented, based on the report of Peng et al. Aligned MWCNT sheets can be retrieved from arrays with widths ranging from millimeters to centimeters and a thickness of 20 nm. The MWCNT sheet is ultrathin; hence, silicon can be uniformly coated on the surface of individual MWCNTs. The thickness can be controlled by varying the sputtering time. Silicon is deposited on the MWCNT sheet via the atomic layer deposition method, following which a twisting treatment is performed to realize a flexible composite fiber electrode. Flexible conjugated fibers can be solidly wound with other fibers; MWCNT/Si conjugated fibers are used as active electrodes to produce LIB in which the Li wire is utilized as the reference electrode (Figure 4a). The specific capacity of this battery remains at 1460 mAh∙g^−1^ after bending for 100 cycles, compared with the 1548-mAh∙g^−1^ capacity before bending of the half-cell fabricated from aligned MWCNT/Si composite fibers with a Si weight percentage of 38.1% (Figure 4b). It was found that the half-cell capacity after bending for 100 cycles could be maintained at 80% in 20 cycles at 2000 mAh∙g^−1^ (Figure 4c). The twisted structure in the half-cell has been well maintained during and after bending [53]. 

#### 2.2.4. Origami Configuration

Origami-based approaches represent another alternative for affording better deformability over existing methods that use flexible materials and mechanically designed structures. Using origami, the ancient art of paper folding, compact deformable 3D structures can be created from 2D sheets folded to considerable extents along predefined creases. The origami battery is manufactured by slurry coating electrodes on paper collectors, packaging them with standard materials, and then folding them using the Miura pattern.

Song et al. fabricated the origami battery with LCO and LTO as cathode and anode electrodes, respectively [54]. Moreover, to achieve good foldability and electrical conductivity at the creases after cyclic folding and unfolding, the CNT coated paper collectors are adopted as current collectors. In Figure 4d, the multilayer structure of conventional LIBs in the planar state and origami LIBs using Miura folding are compared. The latter uses a CNT-coated paper collector that folds or unfolds in the corrugated area but does not deform the hard surface, affording the ability to survive corrugation and forming an excellent adhesion among the electrodes. Although voids and cracks were observed in the LTO film in the paper collector after twisting and bending were performed more than 100 times, no noticeable delamination was observed in the CNT-coated current collector in the SEM images of LTO and LCO active layers. This behavior can be explained by the porous structure (e.g., interconnected fabrics of CNT-coated branch current collector), which provides a continuous network for electron transport and considerably enhances the bond to the anode and its active material layers. 

## 3. Stretchable Batteries

Stretchable electronics represents today’s cutting-edge electronic techniques [55]. Stretchable electronics are a type of mechanically flexible electronics that can be bent, folded, crumpled, and stretched while sustaining great mechanical strain and levels of performance; this represents the emerging direction towards next-generation flexible electronics [56,57]. Stretchable electronics can not only be bent similarly to flexible electronic devices but also be stretched, deformed, and wrapped onto soft and elastic surfaces, which cannot be accomplished using conventional flexible electronic devices [58,59]. Rapid developments and remarkable achievements have considerably influenced the field of wearable electronic devices, resulting in the persistent demand for stretchable energy storage systems. The noteworthy features of stretchable electronics enable their application on complex non-coplanar surfaces to provide distinct functionalities. In the last decade, the field of stretchable electronics, along with flexible devices, has gained considerable interest. The applications of stretchable electronics (e.g., wearable electronics, electronic papers, smart clothes, electronic skins, displays, smartphones, artificial intelligence, soft robotics, and medical implants) have rapidly broadened [1,14,15,55]. Considerable efforts have been devoted to investigating the unique features of stretchable electronics. Stretchable electronics has established the foundation for the development of stretchable electrodes based on wrinkled metal films on elastomer substrates and initiated the research and development of stretchable electronics [60,61,62]. Further research from Rogers research group extended the field of stretchable electronics by integrating semiconductor components with metal electrodes; consequently, stretchable circuits were achieved [63]. Flexible and stretchable electronics may not be able to compete with conventional rigid electronics in terms of device performance because of the lack of capacity to maintain sufficient energy density, the necessity of complicated configurations, the lack of capability to stabilize large strain and shape deformations, and the insufficient cycle life. When the rigid silicon substrate is changed to a plastic substrate, the device performance significantly decreases. In addition, while bending-induced strains (typically to values of ~1% or less) decrease linearly with thickness, stretching-induced strains (≫ 1%) resulting from not only bending but also twisting, stretching, compressing, etc., are not correlated with thickness [64]. To resolve these various problems, two common strategies are adopted: (1) the use of materials with intrinsic stretchable features; (2) the formulation of a structural design that can operate under mechanical strain. In the following sections, the recent progress on these two strategies is introduced. 

### 3.1. Stretchable Strategies: Materials

One of the common strategies to achieve stretchable electronics is the use of stretchable materials, which intrinsically provide a direct approach to attain higher mechanical robustness, higher device density, and scalable fabrication [65]. 

#### 3.1.1. Polydimethylsiloxane (PDMS)

Among the recognized stretchable substrate materials appropriate for the fabrication of implantable electronic systems, polydimethylsiloxane (PDMS) is one of the most promising because of its specific mechanical properties (compliance and softness), good biocompatibility, and thermal stability [66,67]. It has been frequently utilized as a substrate, scaffold, or transfer medium for flexible and stretchable electronics because of the foregoing advantages. 

**Figure 5:**
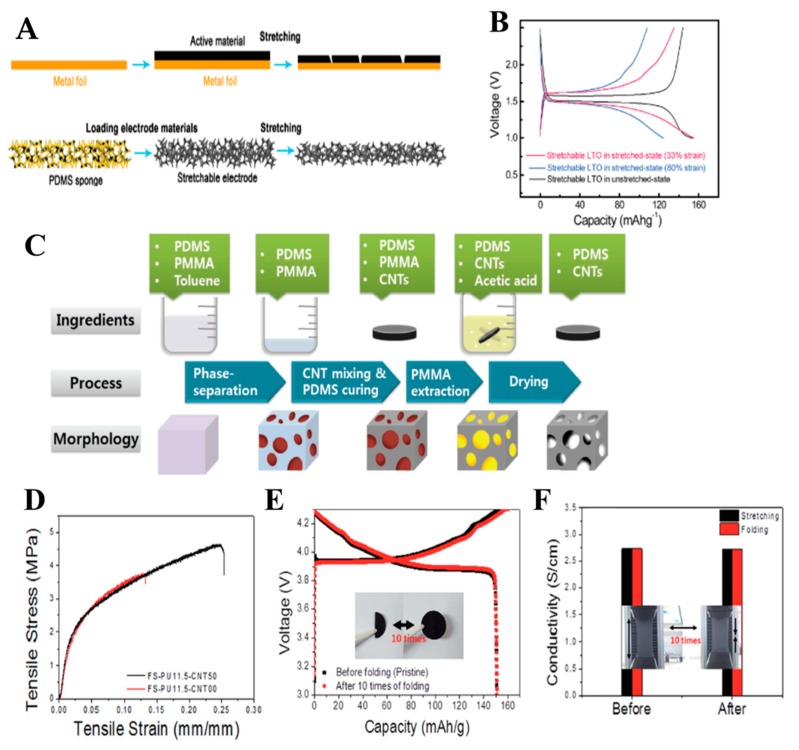
Electrochemical performance and stretchability of the stretchable electrodes. (**A**) Schematic illustration for the comparison of the conventional electrode using metal foil and the stretchable electrode based on the PDMS sponge. Charge/discharge voltage profiles of the stretchable. (**B**) Comparison of charge/discharge voltage profiles between the stretchable LTO anode in unstretched state and stretched states with various strains. Adapted with permission from [68]. Copyright (2016) *WILEY-VCH Verlag GmbH and Co. KGaA, Weinheim.* (**C**) The procedure to fabricate the porous PDMS–CNT nanocomposites. Adapted with permission from [6]. Copyright (2012) *WILEY-VCH Verlag GmbH and* *Co. KGaA, Weinheim.* (**D**) Stress–strain curves of FS-PU11.5-CNT50 and FS-PU11.5-CNT00. (**E**) Charge/discharge capacity after 10 rounds of folding/unfolding and (**F**) electronic conductivity after 10 rounds of folding/unfolding or stretching. Adapted with permission from [76]. Copyright (2017) *The Royal Society of Chemistry*.

Liu et al. reported a novel and simple method to fabricate stretchable electrodes using highly elastic 3D porous sponge-like PDMS scaffolds [68]. Sugar cubes are used as pore-creating agents for fabricating stretchable 3D porous sponge-like PDMS scaffolds. Inexpensive and readily available, sugar with controlled grain size as a template is extremely effective for the fabrication of porous materials. Moreover, sugar can be easily removed simply by dissolving it in water and then reused after evaporating the water. In this work, Li_4_Ti_5_O_12_ (LTO) anode and LiFePO_4_ (LFP) cathode are used as examples to demonstrate the performances and feasibility of stretchable electrodes based on the obtained PDMS sponges. The deformability of stretchable electrodes using sponge-like PDMS is illustrated in comparison with the conventional electrode that uses planar metal foil (Figure 5a)). It is highly possible for the conventional electrode to crack and fall off when stretched, indicating that the conventional electrode has insufficient deformability. On the other hand, the obtained stretchable electrode exhibits significant stretchability without cracks. Here, a number of electrochemical measurements are performed on the fabricated electrodes before and after deformation. The specific capacity of conventional LTO electrodes seems to exhibit considerable performance at a low mass loading. As the mass of electrode materials increase, however, the specific capacity significantly decreases. On the contrary, the stretchable electrode exhibits better performance as the mass of the electrode materials increases. It exhibits remarkable electrochemical performance because of the 3D interconnected porous structure not only in the unstretched state but also in the deformed state. Stretchable LTO/LFP electrodes can withstand 80% strain; moreover, with various stretch–release cycles, the LTO anode and LFP cathode exhibit excellent capacity retentions of 82% and 91% after 500 cycles, respectively. To better understand the remarkable electrochemical properties of the stretchable electrode based on the porous sponge-like structure, the charge/discharge voltage profiles for the half cells using the LTO anode in the stretched status at various strains (33% and 80%) are obtained, as shown in Figure 5(b). Compared to the unstretched electrode, the battery with a stretched electrode (33% strain) exhibits capacity retention of up to 94% because of its good ionic and electronic conduction and mechanical property. 

Along with applications exploiting their flexibility, CNTs have been adopted to increase electrical conductivity. The CNTs also form considerably better interconnections because of their one-dimensional structure. A stretchable CNT/ PDMS composite electrode has been prepared using porous PDMS. Chemical vapor deposition is used to directly attach the CNTs on the PDMS surface [69]. Lee et al. introduced a new approach based on the morphological optimization of highly porous CNT/PDMS nanocomposite electrode for stretchable lithium-ion batteries [6]. In their study, the CNT/PDMS composite is obtained from the phase separation of poly(methyl methacrylate) (PMMA) in PDMS; the PMMA is removed to obtain well-controlled pore networks (Figure 5c). The concentration and dispersion of CNTs and fractions and sizes of pores are the key factors that affect the degree of percolation, which in turn influence their electrical properties [70]. The capacities significantly vary depending on the scale and fraction of the pores. The pore size is controlled by adjusting the degree of phase separation between the PDMS and PMMA. If the pore sizes are small and the pore volume fractions are high, the CNTs cannot maintain their linear shapes because their lengths (~15–20 μm) are larger than the spaces between the pores. On the contrary, the formation of larger pores increases the pore-to-pore spacing, thereby leading to decreased percolation effect. The moderate pore diameter is therefore 30–50 μm. The optimized porous electrode exhibits a 190 mAh∙g^−1^ capacity, which is 670% higher than that of the nonporous electrode. Porosity-optimized nanocomposite engineering will thus be considerably useful for more practical flexible energy storage systems.

#### 3.1.2. Polyurethane (PU)

It has been reported that the distinct features of polyurethane (PU) render it suitable for use as gel polymer electrolyte, binder, and separator because of its unique chemical and polymeric structure [71,72,73,74,75]. Park et al. reported free-standing electrodes, which use a PU binder and LCO active material, for highly deformable LIBs [76]. The PU binder is tested with a PVDF binder in order to compare their characteristics. Compared with the PVDF, the PU exhibits a higher adhesive strength of approximately 0.5 N, which can enhance its adhesive properties to be used as a polymeric binder for LIB electrodes. The initial charge and discharge capacities at various C-rates and cycling stability after 100 cycles show insignificant differences between the PU and PVDF. In this study, Park et al. also compared the tensile strength and electronic conductivity of free-standing electrodes. The deformability of the fabricated electrode was highly improved by adding MWCNTs. As presented in Figure 5d, the elongation increased from 13.1% to 24.9%, indicating that the addition of MWCNTs to the free-standing electrode doubled the tensile strength of the electrode at the maximum load. Moreover, the rate capability of these electrodes remarkably improved because of the increased electronic conductivity induced by MWCNT addition. The obtained electrodes were then tested through repetitive folding without any capacity loss observed. After 10 rounds of folding or stretching, the electronic conductivity of the free-standing electrode exhibited an insignificant electronic conductivity difference (Figure 5e,f). 

#### 3.1.3. The Other Materials

In many instances, flexible devices are generally fabricated by design modifications and elastic substrates that are intrinsically stretchable. Along with generally used materials illustrated above, various intrinsically flexible materials, natural rubber (NR), styrene butadiene rubber (SBR), Ecoflex, ethylene-propylene-diene monomer (EPDM), thermoplastic polyurethane (TPU), etc., can reversibly withstand severe deformations (>200%) [77]. In recent years, there has been great interest in lithium-air batteries due to their high theoretical energy density of about 3500 Wh kg^−1^, which is superior to conventional Li-ion batteries. Wang et al. recently developed a stretchable Li-air battery that exhibits high electrochemical performance and deformability, which is granted by intrinsically stretchable Ecoflex substrate [78]. A stretchable air electrode was fabricated by injecting a precursor solution of ecoflex into a template, followed by a stacking rippled CNT array. Cu current collector, punched Ecoflex, and Li sheets were stacked up to fabricate a stretchable Li-air electrode. By stacking gel electrolyte between the air electrode and stretchable Li array electrode, a stretchable Li-air battery was successfully fabricated. Within a strain up to 100%, the fabricated stretchable Li-air battery showed no changes in discharge voltage plateau. Furthermore, the resistance of stretchable Li-air battery remained almost unchanged under severe deformations.

### 3.2. Stretchable Strategies: Designs

Metals are definitely the materials of choice for a majority of existing nanoelectronic devices. When fabricating stretchable devices, however, it is considerably important to make new designs to impart stretchability to rigid metal films that generally can only withstand a strain of <1% [64]. In developing stretchable electronics, few design strategies have been employed by different researchers over the years. Several major designs include the wavy shape (buckled or wrinkled); wire shape; textile shape; serpentine bridge–island; and kirigami and origami-inspired designs [79]. In the following sections, these strategies are described schematically. 

#### 3.2.1. Wavy-Shape Configuration

The wavy shape structure has been widely used because of its simple fabrication process and capability of attaining high stretchability [57,80,81,82,83,84]. The relaxation of a thin layer of active materials bonded on a pre-strained substrate can form a wavy shape structure [1,85,86,87]. The three following steps are used for designing the wavy shape structure: (1) pre-straining of an elastomer substrate, (2) transfer of active electrode materials onto the pre-strained substrate, and (3) release of pre-strain in the substrate.

**Figure 6:**
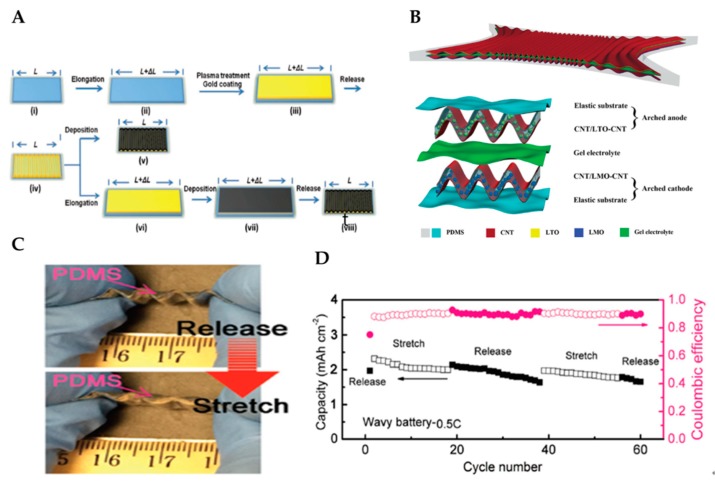
(**A**) Schematic procedures used to prepare buckled Au or PPy-pTS films on SIBS substrate and FESEM images of these products. Buckled PPy-pTS fabrication comprises surface treatment, Au sputter coating, relaxation of the pre-strained SIBS substrate and PPy electrodeposition. 2D buckled PPy-pTS film was electrodeposited on the re-elongated Au coated substrate, obtained after the relaxation of the substrate. Adapted with permission from [67]. Copyright (2011) *WILEY-VCH Verlag GmbH and Co. KGaA, Weinheim* (**B**) schematic illustration of a super-stretchy battery and its multilayered structure. Adapted with permission from [84]. Copyright (2015) *Adv. Mater*. Electrochemical performances at the dynamic state of the wavy battery. (**C**) Photographs showing that when stretching the wavy battery, PDMS was stretched to accommodate the deformation. (**D**) Cycling performance and Coulombic efficiency for the wavy battery under releasing and stretching states (50% strain). Adapted with permission from [91]. Copyright (2017) *WILEY-VCH Verlag GmbH* *and* *Co. KGaA, Weinheim*

Wang et al. reported the fabrication of buckled Au films on PDMS substrates for Mg battery applications [67]. The Au is a common material used to fabricate stretchable electronics by depositing it on an elastic substrate and forming a wave-like structure after the relaxation of the pre-strained substrate. In their study, the PDMS substrates were pre-stretched before the Au film deposition and then released after the deposition [88,89]. The schematic procedures used to prepare buckled Au films are illustrated in Figure 6a. Product v) was fabricated by directly depositing PPy–pTS (polypyrrole, containing dopant p-toluenesulfonate anion) on an obtained buckled Au-coated substrate. Product ⅷ) was then fabricated by depositing PPy-pTS after the elongation of SIBS. Product ⅷ) has achieved larger and trivial buckles and a 2D buckled structure. When the strain was re-applied to the Au-coated stamp (iv) along the previous elongation direction, the Au film was compressed to form trivial buckles. After relaxation, PPy–pTS (vii) was compressed, and major buckles were formed without trivial cracks because of the induced strain during the relaxation process accommodated by the trivial buckles formed; product v) showed noticeable cracks. Wang et al. assessed the electrical resistance of wave-like Au or PPy–pTS films under mechanical strain. The 2D buckled PPy-pTS film can withstand a considerably higher strain compared with the buckled PPy-pTS because of its distinctive structure. Moreover, the 2D buckled PPy–pTS film exhibited remarkable stretchability. It could maintain its electrochemical and mechanical performances during 2000 stretching cycles with 30% strain applied at a fast 5%/s elongation rate without electrical failure, exhibiting its potential application as a stretchable conductor. The electrode is stable when re-stretched to its pre-strain, but cracks may appear upon further stretching. Wang et al. showed that the Au film can maintain its integrity up to a 70% strain. The fabricated material can retain its electrochemical properties in Mg batteries after 2000 stretching cycles with a 30% applied strain. Combining both superior stretchability and electrochemical properties make it a potential material for stretchable electronics. 

Weng et al. made a report on the advanced research of wavy-shaped highly stretchable LIBs [84]. They created a novel arched structure that can be used to form electrodes with high stretchability and stability under deformation. The battery consists of an arched anode, a gel electrolyte, and an arched cathode. Here, two aligned CNT sheets simultaneously function as the skeleton and current collector, as illustrated in Figure 6b. Moreover, the capacity exhibited insignificant changes after 500 stretching cycles with a strain of 400%. A super-stretchable LIB was then fabricated by schematically stacking the wavy-shaped cathode, gel electrolyte, and wavy-shaped anode. The battery performance was first analyzed under stretching. Within a 400% stretching, the output energy exhibited insignificant changes. It remained at 97% of the original level even after the fabricated battery has been stretched 200 times to a strain of 400%. As a result, the sandwich structure is found to be a considerably effective approach to anchor LTO or LMO nanoparticles onto wave-like CNT sheets, thereby affording a stable electrochemical performance. The arched structure allows the successful accommodation of large strains that occur when the composite is buckled, resulting in high stretchability. Moreover, the unique hierarchical structure effectively transports electrons and lithium ions to achieve good electrochemical properties. The attained results suggest that the fabricated LIBs have great potential as highly stretchable LIBs. 

Despite the progress on stretchable electrodes, one of the most difficult challenges for stretchable batteries is having stretchable packaging. Stretching can lead to internal structure changes, which can easily induce leakage. Although several investigations have been performed recently, packaging remains a problem. Liu et al. fabricated full stretchable LIBs with LiCoO2 (LCO) as cathode and graphite as an anode [87]. This battery is based on a wavy shape where all the components, including cathode, anode, separator, current collectors, and even packaging, can be stretched entirely. Two major novelties are important in this battery: (1) the stretchable sticky separator and (2) the device-scaled wavy structure. The electrospun polyurethane/poly(vinyl idenefluoride)(PU/PVDF) membrane is introduced as a separator. This allows the electrodes to stick together and imparts good ion contact for the battery, particularly at the dynamic state. The PDMS filled in the valley region affords capacities for reversible tension and compression deformations. Uniformly formed pores provide high lithium-ion transport and excellent electrochemical performances. Liu et al. demonstrated the electrochemical properties of the fabricated battery using light-emitting diode under a 50% strain, as shown in Figure 6c. Moreover, the long-term charge/discharge cycling of the battery at a 0.5-C rate in a dynamic state was conducted, as shown in Figure 6d. The first charge/discharge cycle for the wavy-shaped battery was performed at a released state, and the battery was then run at alternates of stretched (50% strain) and released states until 60 cycles. The results demonstrate that the battery process has a highly stable electrochemical performance at the dynamic state of repeated release/stretch cycles. The high areal capacity of approximately 1.65 mAh∙cm^−2^ at the 60th cycle under a released state was achieved with a capacity retention of 85%, which is a considerable performance [64]. The capacity reduction is caused by the edge effect, which could be reduced by using an anode with a larger area than the cathode in the cell assembly. 

The introduction of the polymer binder is common and necessary when attaching substrates and active materials. However, polymer binder degrades the ion transport capability and restrains the electrical conductivity, thereby leading to a significant capacity loss [90]. It is therefore highly desirable to accommodate binder-free electrodes that will not only significantly improve the specific capacity but also solve the cycling degradation problem introduced by the stretching-releasing process. Gu et al. [90] fabricated a stretchable cathode material for lithium-ion batteries by the in situ growth of LiMn_2_O_4_ nanocrystals into buckled CNT films. The low temperature and annealing-free synthesis environment are also cost-effective and reduce surplus reaction steps. In addition, the buckled electrode made of LMO/CNT films with flexible PDMS substrates significantly enhances stretchability. In comparison with the conventional electrode preparation method, the reported binder-free 3D structure inhibits particle aggregation and nonuniform distribution. Moreover, the large surface area and 3D structure enable the efficient electrolyte infiltration and confinement of Mn^3+/4+^ ions from dissolving into the electrolyte during the Li^+^ reversible intercalation processes. 

#### 3.2.2. Kirigami and Origami Configuration

Origami and kirigami are ancient techniques for making paper works of art by folding or cutting 2D sheets to create 3D objects, which can achieve a high level of stretchability. Origami-based approaches can be potential alternatives for enabling better deformability over existing methods that use elastomeric materials and mechanically designed structures (e.g., buckling and serpentine shapes) [92]. Origami-based approaches, however, involve two problems that have to be solved. First, the deformability of the origami-based devices is limited to folding or bending. Second, the folded state involves uneven surfaces, which can cause inconvenience when integrated with planar systems. On the contrary, kirigami LIBs exhibit excellent mechanical and electrochemical performances under various deformations. Song et al [93]. recently demonstrated a stretchable kirigami LIB that can be deformed at an unprecedented high level, including folding, bending, twisting, and stretching. Kirigami is a variant of origami that exploits additional degrees of freedom afforded by combining cutting and folding to expand the range of stretchability [94]. Three kirigami patterns, namely, the zigzag-cut pattern, cut-N-twist pattern, and cut-N-shear pattern, have been designed to fabricate stretchable LIBs. The LIB under its fully stretched state is presented, showing that the stretchability of the fabricated LIB could reach more than 100%. This kirigami LIB exhibited good and stable electrochemical performances under extreme deformations. The capacity retention of more than 85% and Coulombic efficiency of 99.8% over 100 cycles could be achieved for the kirigami LIB under alternative states of compact and stretched states. These concepts can potentially open important research frontiers and create unusual engineering applications in other areas, such as aerospace, mechanical, and civil engineering, for studies in reconfigurable structures, broadband energy harvesters, and many others. 

#### 3.2.3. Serpentine Bridge-Island Configuration

Elastically stretchable electronics based on elastomeric substrates could diminish the gap between traditional rigid electronic devices and soft curvilinear environment [13]. These devices and systems are of great interest because of their ability to provide applications that cannot be achieved using conventional technologies [13]. When implemented with advanced materials and mechanical designs, such devices can be bent, twisted, and stretched without mechanical fatigue or any significant change in operating characteristics, even when brittle materials are used [57,95,96,97]. Many of these systems exploit island–bridge architectures, where the active components reside at the islands, and electrical interconnects form the bridges; the latter are the key features for stretchability. In general, stretchable devices must accommodate two competing design goals. First, stretchable devices must achieve a high surface filling ratio, which requires a large coverage of islands. Second, stretchable devices must impart high mechanical stretchability, which typically demands long bridges between these islands [13]. The serpentine interconnect represents an advanced epitome, with progressive stretchability for a given spacing between adjacent islands.

**Figure 7:**
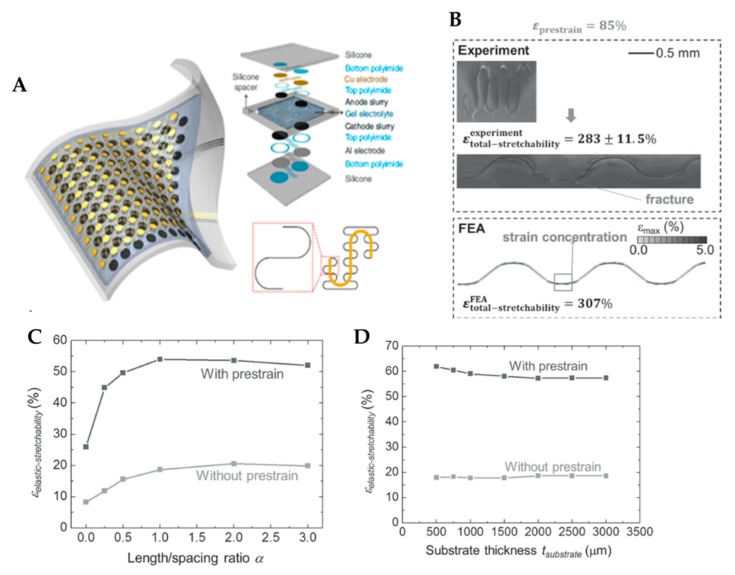
(**A**) Schematic illustration of a completed device, in a state of stretching and bending. Adapted with permission from [64]. Copyright (2013) *Macmillan.* (**B**) Experimental image of the serpentine interconnect and the fracture sites due to cyclic stretching (with an amplitude of 185%), and FEA results for the strain distribution when stretched to the predicted elastic stretchability (189%), for the case of 85% prestrain. Adapted with permission from [98]. Copyright (2013) *WILEY-VCH Verlag GmbH and Co. KGaA, Weinheim*. The influences of geometric parameters ((**C**) for the length/spacing ratio, (**D**) for substrate thickness) on the elastic stretchability of serpentine interconnects with and without prestrain. Adapted with permission from [98]. Copyright (2013) *WILEY-VCH Verlag GmbH and Co. KGaA, Weinheim.*

A work from Rogers research group provided a significant impact in the field of stretchable batteries. They schemed a segmented design in the active electrode materials, with unusual “self-similar” interconnecting structures between them, using silicone elastomers as substrate (Figure 7a) [64]. The figure below shows pouch cells, which consist of arrays of small-scale storage components connected by conductive frameworks with a highly stretchable feature. The current collectors, consisting of patterned circular aluminum and copper disks, were first fabricated by photolithography; the LCO and LTO were used as electrodes herein. A square array of 100 disks of an electrode connected in parallel was covered by elastomer sheets, resulting in a capacity density of ≈1.1 mAh∙cm^−2^ even under a biaxial strain of up to 300%. Moreover, a gel electrolyte injected into the gap provides media for ionic transport. The thin encapsulation layer of an acryloxy perfluoropoly-ether elastomer as package material allows the whole device to achieve a reversible stretchability of up to 300%. Moreover, the insignificant decrease in capacity can be achieved in the lithium-ion battery for up to 20 cycles of considerable recharging. 

Zhang et al. reported a pre-strain strategy with serpentine interconnects on the island–bridge design for stretchable electronics. [98]. Herein, at least two classes of interconnect structures have been proposed: 1) straight ribbons with non-coplanar arc shapes; 2) serpentine traces composed of arcs and straight lines. In the pre-strain strategy, serpentine interconnects are transferred, printed, and bonded onto a stretched substrate. When the strain releases and causes deformations in the interconnect, the stretchability is enhanced. The right frame in Figure 7b shows good cycle stability as there are no cracks observed after the release of pre-strain for 25,000 cycles. The electrical resistance with a four-point probe technique is measured to show the compatibility for devices during various levels of deformation. Figure 7c shows the stretchability and maximum pre-strain for avoiding plastic yielding. When the substrate is considerably thicker than the interconnect, the elastic stretchability sharply increases as the metal thickness decreases from 4.0 to 0.3 μm (Figure 7d). This sharp increase in elastic stretchability is caused by different buckling modes for thin and relatively thick metal layers. As a result, it is confirmed that the large increase in elastic stretchability is caused by the pre-strain. In particular, the elastic stretchability exceeds 100% for extremely thin metal (t metal < 0.45 μm). 

#### 3.2.4. Wire and Textile Configuration

To satisfy the flexible requirement of portable and wearable electronics, developing a wire-shaped lithium-ion full micro-battery is critical and remains a problem. The wire-shaped micro-device can be easily woven into a flexible textile that has been proposed to represent an effective route to overcome the disadvantages of conventional batteries, e.g., the difficulty of application caused by the stretching problem. Inferior performances, including low energy densities, also prevented them from appearing in the market. Lithium-ion batteries with significantly higher energy densities have therefore gained considerable interest. Although the lithium-ion battery has excellent properties, critical safety problems hinder it from being accommodated on flexible electronic devices. The safety problems associated with dendrite growth on the anode surface will lead to short circuit and heat release, which may result in combustion. 

**Figure 8:**
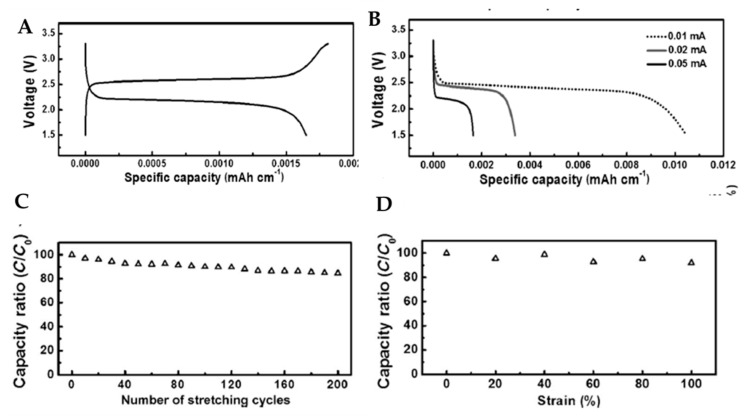
Electrochemical performance of the wire-shaped full cell with a length of 1 cm. (**A**) Galvanostatic charge and discharge curves at 0.05 mA. (**B**) Discharge profiles at different currents. (**C**) Dependence of the specific capacity on the strain. (**D**) Dependence of the specific capacity on the number of stretching cycles with a strain of 100%. C0 and C correspond to the specific capacities before and after stretch. Adapted with permission from [99]. Copyright (2014) *Wiley-VCH Verlag GmbH and Co. KGaA, Weinheim*.

To solve this problem of dendritic lithium on the anode, Ren et al. fabricated novel and safe wire-shaped lithium-ion batteries with embracing LTO and LMO nanoparticles into two aligned multi-walled carbon nanotube (MWCNT) yarns that serve as anode and cathode, respectively [99] The MWCNT has been adopted for its various features, such as aligned nanostructure and high electrical conductivity; no binder and current collector are also necessary. Aligned MWCNT/LTO and MWCNT/LMO composite yarn electrodes are prepared by coating the LTO and LMO nanoparticles onto the MWCNT sheet; then, the resulting composite films are twisted. The fabricated battery exhibited a remarkable electrochemical performance. The alignment is advantageous in extending the excellent mechanical and electronic properties of individual MWCNTs to a macroscopic scale. The MWCNTs function as effective pathways for charge transport and serve as current collectors, which provide remarkable electrochemical properties to composite yarns. The electrochemical performances are verified, as shown in Figure 8. Figure 8a is a typical voltage profile of charge and discharge at a current of 0.05 mA that exhibits a reversible capacity of 70 mAh∙g^−1^. Moreover, the discharge plateau voltage slightly decreases from 2.5 to 2.2 V with increasing current densities (Figure 8b); this clearly shows that the wire-shaped battery can stably operate at high current densities. The electrochemical performance of the stretchable lithium-ion battery is further demonstrated under increasing elongations from 0, 20, 40, 60, and 80, to 100%. As a result, the specific capacity exhibits a slight loss when the battery is stretched by 100% (Figure 8c) After 200 cycles of 100% stretching, the specific capacity remains more than 80% (Figure 8d). The maximum strain in the battery depends on the stretchability of the substrate; a strain of up to 600% could be achieved with an elastic polydimethylsiloxane (PDMS) substrate. Details of this investigation are introduced next.

To overcome the disadvantages of conventional batteries, Zhang et al. [53] fabricated super-stretchable fiber-shaped lithium-ion batteries with MWCNT/LiMn_2_O_4_ as a cathode and MWCNT/Li_4_Ti_5_O_12_ as an anode. These electrodes are then wound onto an elastic substrate to form a spring-like structure; this structure is further anchored by the coated gel electrolyte. No evident destruction in the structure is observed for the two fiber composite electrodes after stretching by 200%. The electrochemical properties of the fiber-shaped lithium-ion battery are further demonstrated. The shapes of the charge/discharge curves are well-maintained with increasing strains from 0%, 50%, 100%, and 150% to 200% at a current density of 0.1 mA∙cm^−1^. Moreover, no obvious decrease in capacity is observed, and the specific capacity of more than 93% is maintained at a strain of 200%. These results demonstrate that the fiber-shaped lithium-ion battery exhibits comparable performance to non-stretchable and stretchable counterpart planar batteries; this may be attributed to the twisted structure of fiber electrodes and the enhanced stretchability caused by the flexible substrate and gel electrolyte. Furthermore, the obtained fiber-shaped batteries can be effectively scaled up and well-accommodated with the folding and stretching of a bracelet and knitted sweater. As a result of the foregoing remarkable results, the fabricated batteries seem to have potential applications in wearable electronics.

## 4. Conclusions and Perspectives

Flexible energy storage devices have been extensively applied. The fabrication process of energy devices and the world’s present lifestyle have considerably changed because of the development of flexible electronics. Although this review showed considerable performances of bending and folding batteries, limited applicable angle and strain remain challenges for the bending and folding energy storage devices to be actually applied to skin attachable devices and medical implants. Thus, there is a long way to go until the commercialization of bending and folding batteries in practical applications, and more effort is required. In the last decade, along with an effort to develop bendable and foldable batteries, the developments of stretchable batteries have blossomed into one of the fields many researchers are engaged in. This review illustrates the progress of flexible and stretchable batteries. While describing the features of flexible energy storage systems, the progress and development achieved in materials exploration and structural designs are emphasized. As the demands for thin and high-performance film significantly increased, considerable efforts have also been expended to obtain a suitable binder and perform device integration. Moreover, several synthetic methods have been established to synthesize high-quality materials, including chemical and physical strategies. There remain several problems that have to be resolved for improving the utilization of these materials and strategies for flexible devices. They can improve the electrochemical performances of materials depending on the distribution, density, types of chemical bonds, and three-dimensional arrangement of the composite material. Furthermore, it is critical to developing large-scale and industrial production techniques to produce reliable materials with improved electrochemical and mechanical performances while reducing manufacturing costs. 

To afford extreme deformability to flexible energy storage devices, it is considerably important to fabricate devices with intrinsically flexible materials or introduce effective structural design configurations. Intrinsically flexible materials provide a direct route to achieve flexible devices with higher mechanical robustness and device density. Although the foregoing efforts have been devoted, numerous problems still remain to be resolved, such as affording further bendability and stretchability. Modifying the device’s structure by various structural designs is therefore crucial. The following research aspects should be launched to overcome the current drawbacks and further improve the performance of flexible energy storage devices. 

Despite remarkable progress, flexible LIBs still encounter unwanted cracks under complex deformations, such as twisting. Moreover, the thinness of LIBs, which is one of the unique features of flexible LIBs, can lead to complete breakage under accidental cutting. To solve the foregoing problems, various research investigations on self-healable electronic devices have been recently investigated in soft electronics and robotics. There are two main strategies to fabricating highly self-healing electrochemical devices: (1) adopting dynamic reversible chemical bonds into conductive polymers; (2) the introduction of composites of polymers and capsules along with healing agents [100,101] Consequently, self-healing polymers heal cracks that could lead to electrochemical performance degradation by reversible chemical bonds [102,103], ligand–metal bonding [104], host–guest interaction [105,106], and hydrogen bonding [107,108]. In the pioneering work of Zhao et al. [109], self-healable electrodes are fabricated by introducing self-healing polymer with aligned CNT and gel electrolyte. In this study, LiMn_2_O_4_ (LMO) and LiTi_2_(PO_4_)_3_ (LTP) nanoparticles serve as active materials that are uniformly dispersed and attached to the aligned CNTs. The polymer substrates consist of supramolecular polymer networks that are rich in multiple hydrogen bonds, which can self-reconstruct after breaking. During experiments, it is clearly observable that the fabricated electrodes have fully recovered to initial states after severe deformations, such as bending, stretching, twisting, and even cutting. Moreover, the self-healing electrodes exhibit outstanding electrochemical and mechanical self-healing performances. The fabricated self-healing LIBs, therefore, have considerable potential for next-generation flexible energy storage devices. Yet, there still remain challenges, such as combining fast and reproducible healing properties, high conductivity, simple processing in terms of skin attachable devices, and biocompatibility [110]. Hopefully, further investigations and optimization on the electrochemical and physical performances of these self-healing devices will promote their uses toward various applications. 

All-solid-state lithium-ion batteries are potential candidates for overcoming the safety and energy limitations of common lithium-ion batteries [111]. Replacing the liquid electrolyte with a solid one provides substantially improved safety. In addition, to realize the safety and flexibility of batteries with high performances and benign mechanical properties, exploring solid-state Li-ion electrolytes with freedom design is strongly preferred. Traditional all-solid-state LIBs use a rigid substrate, and its replacement with a flexible substrate can endow outstanding deformability to devices [112]. A bendable all-solid-state LIB was introduced by Koo et al. based on a thin layer of lithium phosphorus oxynitride (LiPON) with a thickness of approximately 2 μm as the solid electrolyte [113]. All components, including current collector, LCO cathode, LiPON electrolyte, and Li metal are then deposited on a mica substrate, and the multilayer is then peeled off using sticky tapes and transferred onto poly(dimethylsiloxane) (PDMS) sheet. This bendable thin-film LIB exhibits the highest charging voltage of 4.2 V and capacity of 106 μAh∙cm^−2^ at a non-bending status; it decreases to 99 μAh∙cm^−2^ at a bending radius of R = 3.1 mm. 

After decades of development, flexible energy storage systems have opened up opportunities for many interdisciplinary research fields and rapidly expanded and enriched them. Although numerous worldwide scientific works have offered abundant and remarkable results for materials, mechanics, design, and initial applications of flexible and stretchable electronics, there remains vast opportunity for exploration and construction in this field.
